# The Association between Urinary Polycyclic Aromatic Hydrocarbons Metabolites and Type 2 Diabetes Mellitus

**DOI:** 10.3390/ijerph19137605

**Published:** 2022-06-22

**Authors:** Xue Wang, Ang Li, Qun Xu

**Affiliations:** 1 Department of Allergy & Clinical Immunology, National Clinical Research Center for Immunologic Diseases, Peking Union Medical College Hospital, Chinese Academy of Medical Sciences, Peking Union Medical College, Beijing 100730, China; pumc_wangxue@student.pumc.edu.cn; 2 Department of Epidemiology and Biostatistics, Institute of Basic Medical Sciences Chinese Academy of Medical Sciences, School of Basic Medicine Peking Union Medical College, Beijing 100005, China; pumcleon@ibms.pumc.edu.cn; 3 Environmental and Health Sciences, Chinese Academy of Medical Sciences, Peking Union Medical College, Beijing 100005, China

**Keywords:** polycyclic aromatic hydrocarbons (PAHs), mono-hydroxylated PAHs (OH-PAHs), environmental pollutants, diabetes

## Abstract

Polycyclic aromatic hydrocarbons (PAHs) are considered to be endocrine disruptors. In this study, the evidence on the association between PAHs and diabetes was systematically reviewed. PubMed, EMBASE, and ISI Web of Science were systematically searched for studies reporting the association between PAHs and diabetes. Of the 698 articles identified through the search, nine cross-sectional studies were included. Seven were conducted in the general population and two in coke oven workers. Fixed-effects and random-effects models were used to calculate the total effect. Subgroup analysis was further carried out according to the types of PAH metabolites. The results showed that the odds of diabetes were significantly higher for the highest category of urinary naphthalene (NAP), fluorine (FLU), phenanthrene (PHEN), and total mono-hydroxylated (OH-PAH) metabolites compared to the lowest category. The pooled odds ratios (OR) and 95% confidence intervals (CI) were 1.52 (95%CI: 1.19, 1.94), 1.53 (95%CI: 1.36, 1.71), 1.43 (95%CI: 1.28, 1.60), and 1.49 (95%CI: 1.07, 2.08), respectively. In coke oven workers, 4-hydroxyphenanthrene (4-OHPh) was significantly correlated with an increased risk of diabetes. Exposure measurements, outcome definitions, and adjustment for confounders were heterogeneous between studies. The results of the current study demonstrate a potentially adverse effect of PAHs on diabetes. Further mechanistic studies and longitudinal studies are needed to confirm whether PAH metabolite levels are causative, and hence associative, with increased diabetes incidences.

## 1. Introduction

Ambient air pollution exposure increases human morbidity and mortality [1] and is a leading contributor to the global burden of disease [2]. Polycyclic aromatic hydrocarbons (PAHs), which are products formed during incomplete combustion of fossil fuels and cigarette smoking [3], are often detected in the environment and are regarded as endocrine disruptors [4]. The harmful effects of PAHs on human health depend on the duration of exposure, the concentration and toxicity of PAHs, and the route of exposure. Humans are exposed to PAH through multiple routes, including consumption of food and water, inhalation of air, resuspended soil and dust, and dermal contact with soil and dust. The existence of various routes of exposure makes it difficult to assess the adverse health effects of PAHs. Mono-hydroxylated PAHs (OH-PAHs), a class of PAHs biotransformation products in urine, have emerged as useful biomarkers for assessing PAH exposure [5]. The measurement of OH-PAHs concentrations in urine allows assessment of the combined uptake of all exposure routes [6].

Several studies have supported PAHs as a risk factor for type 2 diabetes mellitus (T2DM). The possible pathways include the activation of inflammation [7,8], insulin resistance [9], altered release of cytokines [10], oxidative stress [11], and interference with the endocrine system [12], such as affecting beta cell function [13] and estrogen signaling [4,14]. Everett CJ et al. investigated the association between 9 OH-PAHs and serum C-reactive protein (CRP) and found that 2-hydroxyphenanthrene (2-OHPh) and 9-hydroxyfluorene (9-OHFlu) were associated with elevated CRP [8]. Elevated CRP, in turn, has been reported to be associated with a high risk of T2DM [15]. Guo et al. showed that compared with men, women working near a coke oven displayed significantly higher levels of oxidative stress biomarker, including urinary 8-hydroxy-deoxyguanosine and 8-iso-prostaglandin-F2a [11]. A study conducted by Khalil et al. in high-fat-diet–fed mice showed that chronic exposure to environmental PAHs can increase the risk of T2DM by reducing glucagon-like peptide 1 expression and inducing the production of pro-inflammatory cytokines [7]. Gato et al. reported that rats treated with 2-aminoanthracene (2AA), a PAH found in a variety of products, had a larger mean adipocyte size. Similarly, 2AA-treated rats had higher serum glucose levels compared to the controls [10]. A birth cohort study conducted in Korea also found that maternal exposure to dietary PAHs during pregnancy was associated with decreased birth weight [16], which may increase the risk of T2DM in adults [17]. In addition, occupational studies of coke oven workers have shown that 4-hydroxyphenanthrene (4-OHPh) levels were positively associated with the risk of T2DM [18].

Khosravipour et al. (2020) systematically reviewed the relationship between urinary metabolites of PAHs and diabetes and proposed a significant positive association between urinary levels of PAH metabolites and diabetes [19]. However, since this review, several epidemiological studies have been published, including studies conducted in the general population, as well as studies in occupational workers. The results of the newly published studies are partly inconsistent with previous results. For example, previous studies reported a positive association between 2-hydroxynaphthalene (2-OHNa) and the risk of T2DM [20,21,22], but the study by Lee et al. (2022) reported a significant negative association between high concentrations of 2-OHNa exposure and the risk of T2DM [23]; similarly, previous studies reported a significant positive association between 9-OHFlu and the risk of T2DM [20,24], but Cheng et al. (2021) showed no significant association between the two [25]. The reasons why the results are inconsistent might be due to the locations of the studies, the populations studied, the different levels of pollutants, and the statistical methods implemented. In addition, the meta-analysis by Khosravipour et al. (2020) only included studies conducted in China and the United States [19]. In the current study, we also include original research conducted in Korea, thereby broadening our perspective. Newly published studies may also deepen the understanding of the harmful effects of PAHs. In the current study, by including the latest research, we hope to identify the gaps and limitations in the existing research and offer directions for future research. Furthermore, few existing studies discuss the differences in the effects of PAHs on T2DM in the general and occupational populations. Therefore, in this study, the evidence in the general population and in occupational populations was systematically identified and reviewed. The current study also synthesized these findings to provide policymakers with the best estimate of the effect of PAH exposure on the risk of diabetes.

## 2. Methods

### 2.1. Literature Search Strategy

The PubMed (https://pubmed.ncbi.nlm.nih.gov/), EMBASE (https://www.embase.com), and ISI Web of Science (http://www.webofknowledge.com) databases were systematically searched for eligible studies published up to 24 May 2022, with a combination of terms including “diabetes mellitus”, “diabetes”, “type 2 diabetes”, “T2DM”, “polycyclic aromatic hydrocarbons”, “urinary polycyclic aromatic hydrocarbons”, “polycyclic aromatic hydrocarbons metabolite”, “urinary polycyclic aromatic hydrocarbon biomarkers”, “PAHs”, “hydroxynaphthalene”, “hydroxyfluorene”, “hydroxyphenanthrene”, “hydroxypyrene”, and “hydroxychrysene”. No filters were applied to the study designs. The references from the included publications were scanned again to identify any remaining studies. Titles and abstracts were screened for further decisions, and the full text of eligible articles was downloaded after de-duplication. All the processes were performed independently by the two authors (Xue Wang and Ang Li), and disagreements were resolved through discussion.

### 2.2. Inclusion and Exclusion Criteria

Original studies assessing the relationship between urinary PAH metabolites and diabetes and published in English were included. For eligible studies, the type of urinary PAH metabolites and the definition of diabetes must be clearly stated. Urinary PAH metabolites include at least one of 1-hydroxynaphthalene (1-OHNa), 2-OHNa, 2-hydroxyfluorene (2-OHFlu), 3-hydroxyfluorene (3-OHFlu), 9-OHFlu, 1-hydroxyphenanthrene (1-OHPh), 2-OHPh, 3-hydroxyphenanthrene (3-OHPh), 4-OHPh, 9-hydroxyphenanthrene (9-OHPh), and 1-hydroxypyrene (1-OHP). ΣOH-PAHs represents the sum of PAHs. Diabetes was diagnosed by a physician or based on the use of anti-diabetic medications. Eligible articles were required to quantify the levels of urinary PAH metabolites and to report the association of urinary PAH metabolites with the risk of diabetes. Odds ratios (OR), relative risks (RR), or hazard ratios (HR) and their 95% confidence intervals (CI), should be provided. Studies from different periods of the same cohort were included in the current analysis. Animal studies and studies focusing only on alterations in blood biomarkers (e.g., blood glucose levels and insulin) were excluded. For studies from the same cohort and time period, only the one study with PAHs as the major exposure, with diabetes as the primary outcome, and with the most representative study population was included in the analysis.

### 2.3. Data Extraction

The required parameters were extracted independently by the two authors (Xue Wang and Ang Li). Disagreements were resolved by discussion. The following information was extracted: study location, study period, study design, year of publication, demographics of the participants, definition of exposures and outcomes, and potential confounding factors adjusted for in the statistical model. Effect estimates and their 95% CIs of the association between urinary PAH metabolites levels and the risk of diabetes were extracted in the “final model” or “fully variable adjusted model”. The following methods were used to extract the effect estimates and obtain the necessary raw data: (1) All the included studies presented the odds of diabetes based on the tertile, quartile, or quintile of urinary PAH metabolite levels; therefore, the odds ratio of the highest tertile, quartile, or quintile (Q3, Q4, or Q5) compared with the reference group (Q1) was extracted; (2) If the results in the original study were presented as figures, GetData Graph Digitizer 2.26 was used to extract the data.

### 2.4. Statistical Analysis Methods

Fixed-effect and random-effect models were used to synthesize the associations between urinary PAH metabolites and diabetes. The odds ratio was used as a measure of the association in all recruited studies. The between-study heterogeneity and between-study variance were evaluated by *I*^2^ and τ^2^, respectively. Publication bias was assessed using the Egger’s test and Begg’s test.

Subgroup analysis was performed according to the type of urinary PAH metabolites. The subgroups were set up as follows: (1) Hydroxynaphthalene, including 1-OHNa and 2-OHNa; (2) Hydroxyfluorene, including 2-OHFlu, 3-OHFlu, and 9-OHFlu; (3) Hydroxyphenanthrene, including 1-OHPh, 2-OHPh, 3-OHPh, 4-OHPh, and 9-OHPh; (4) Because 1-OHP is commonly used as a biomarker for exposure to PAHs [26], 1-OHP and ΣOH-PAHs were set as a subgroup.

All analyses were performed with R software (R Foundation for Statistical Computing), version 3.5.0. A two-sided test was used, and *p*-value < 0.05 was considered as statistically significant.

## 3. Results

**Figure 1:**
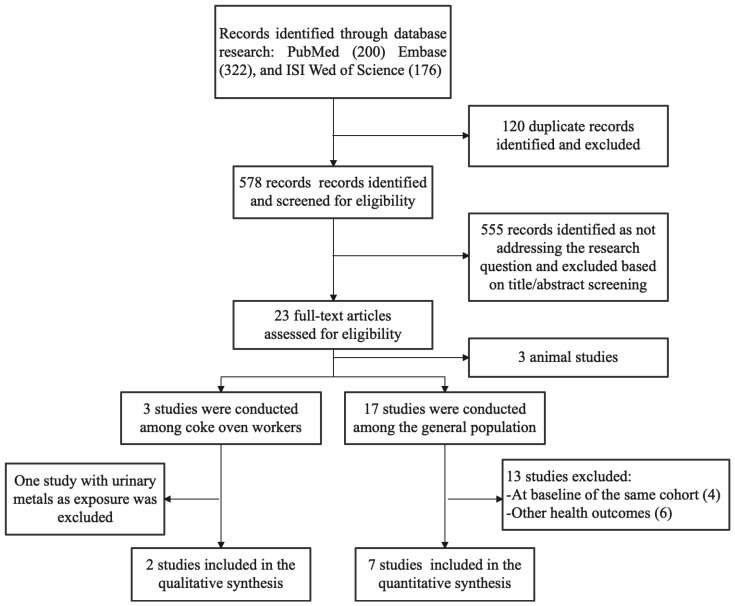
Results of systematic literature search.

The literature selection process is summarized in Figure 1. A total of 698 records were identified through the database search, and 578 records were assessed for eligibility based on the title and abstract after de-duplication. By reading the titles and abstracts, 555 studies that did not investigate the association between PAHs and T2DM were excluded. An in-depth full-text review of the remaining 23 potentially eligible articles was conducted. Seventeen of these studies were conducted in the general population, with an additional three studies conducted in the occupational workers and three studies conducted in animals. Of the 17 studies conducted in the general population, six were from the Wuhan–Zhuhai cohort. Two of the six studies were included in the analysis, one study conducted in April–May 2011 and May 2012 [25], and the other study conducted in April–May 2011 [24]. These two studies were conducted in different time periods and were therefore included in the analysis, whereas the other four studies from the same time period were excluded. Six studies were excluded because they focused on other health outcomes. Of the three studies conducted among the occupational workers, one was excluded because of the use of urine metal as exposure [27]. Finally, nine studies met the inclusion criteria and were recruited in the current analysis. These nine studies were all cross-sectional studies reporting the association between urinary PAH metabolites and the risk of T2DM [18,20,21,22,23,24,25,28,29]. Table 1 shows the details of the nine studies. Three of the studies were conducted in the United States, four in China, and two in Korea.

### 3.1. Potential Bias Due to Outcome Assessment

As shown in Table 1, the definition of T2DM varied slightly among the nine studies. Six studies defined T2DM as abnormal laboratory tests (HbA1c or fasting blood glucose), self-reported physician-diagnosed diabetes, or use of oral hypoglycemic medications or insulin [18,20,21,22,24,25]. One article defined T2DM as self-reported physician-diagnosed diabetes, or use of oral hypoglycemic drugs or insulin [28]. Another study defined T2DM as the use of hypoglycemic medications [23]. In addition, the study conducted by Zhang et al. used metabolic syndrome as the primary outcome [29]. Hyperglycemia, one of the components of metabolic syndrome, was defined in the study as elevated fasting blood glucose or current use of hypoglycemic medications [29]. This definition is similar to the definition of T2DM in other studies. Therefore, the effect of urinary PAH metabolites on hyperglycemia was included in the analysis.

### 3.2. Potential Bias Due to the Exposure Assessment

The included studies used different methods to assess the levels of urinary PAH metabolites in the participants. Gas chromatography–mass spectrometry was the most commonly used method for measuring exposure. The diversity of exposure measurement methods could be a potential source of bias.

The adjustment method for urinary dilution is important when assessing urine PAH metabolite levels. Most of the studies adjusted for urinary creatinine when calculating urinary PAH metabolite concentrations, except for the study conducted by Ranjbar et al., which adjusted for urinary creatinine as a confounding factor in the statistical model [22]. In most cases, urine creatinine was used to adjust for urine dilution. However, urine creatinine is influenced by muscle mass and BMI [30]. Therefore, this method should be used with caution when the study participants have a large BMI. To address this problem, a standardized covariate adjustment method was used in the study conducted by Lee et al. [23]. First, predicted creatinine values were generated for each participant by linear regression analysis. Then the measured creatinine level was divided by the predicted creatinine level to produce a ratio. Finally, the level of the chemical in the urine was divided by this ratio to adjust for urine dilution [29].

### 3.3. Potential Bias Due to Confounder Adjustment

As presented in Table 1, all of the studies were adjusted for the age and sex of the participants, except for one study conducted in coke oven workers which was adjusted for working years rather than age [18]. All of the studies were adjusted for BMI except for the study conducted by Smith et al. [20]. Most of the studies considered confounding effects of socioeconomic status, albeit on different scales. Poverty–income ratio, education, or household income were used as socioeconomic determinants. Smoking and diet contributed to exposure to PAHs [31]. Of the studies, six were adjusted for smoking and three were adjusted for cotinine. Only one study conducted in coke oven workers additionally considered cooking fumes and eating habits [29]. The insufficient control of the source of PAH exposure may increase the risk of bias. In addition, of the two studies conducted in coke oven workers, one adjusted for occupation-related factors (work shift and work sites) [18] while the other did not [29].

### 3.4. Publication Bias

Considering that negative findings may not be published, Begg’s and Egger’s tests were used to examine potential publication bias. No significant publication bias was found for any type of urinary PAH metabolites among the studies conducted in the general population (Figures S1–S4). However, most of the studies detected several types of urinary PAH metabolites, and it is uncertain whether a particular PAH metabolite was detected but not reported, so that a potential publication bias may exist.

### 3.5. Meta-Analysis of the Association between Urinary PAH Metabolites and the Risk of T2DM

**Figure 2:**
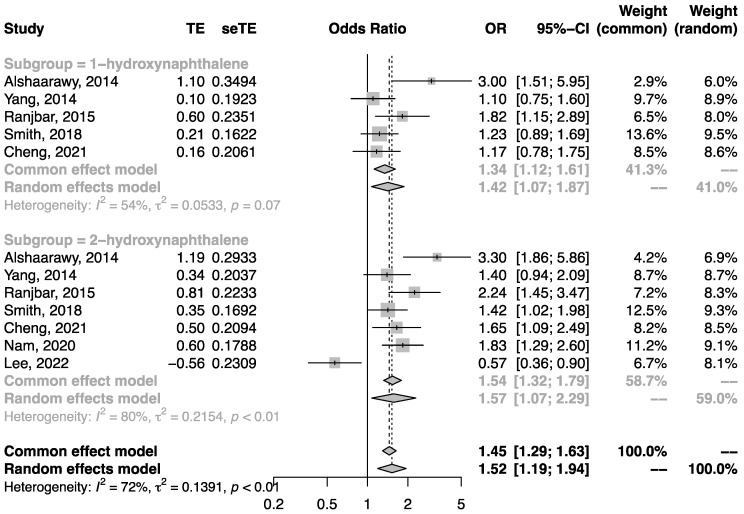
The association between urinary NAP metabolites and risk of T2DM [20,21,22,23,24,25,28].

**Figure 3:**
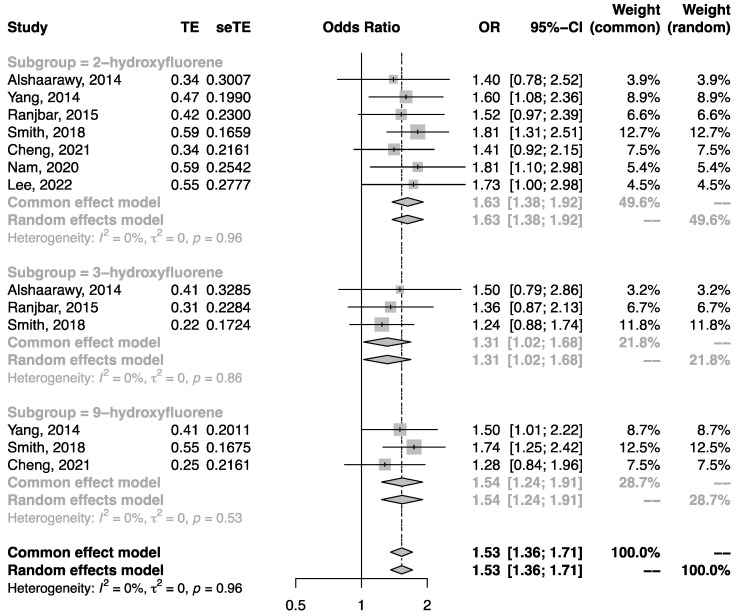
The association between urinary FLU metabolites and risk of T2DM [20,21,22,23,24,25,28].

**Figure 4:**
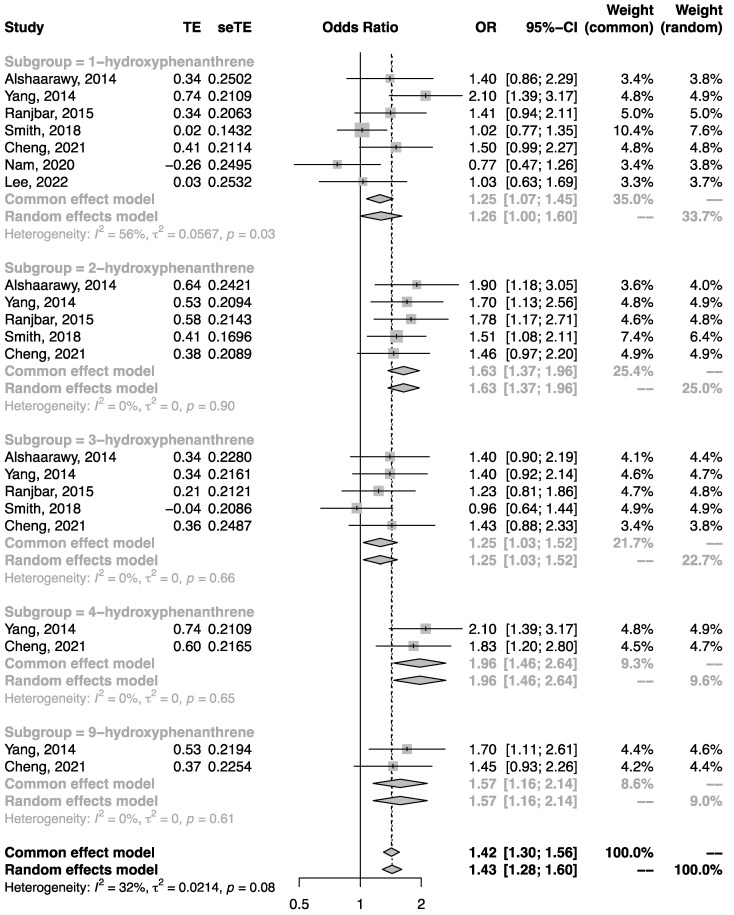
The association between urinary PHEN metabolites and risk of T2DM [20,21,22,23,24,25,28].

**Figure 5:**
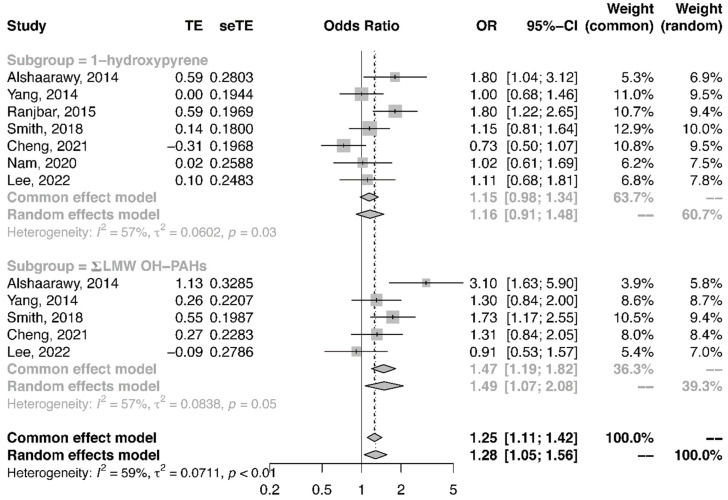
The association between urinary 1-OHP, ΣOH-PAH metabolites, and risk of T2DM [20,21,22,23,24,25,28].

Quantitative synthesis analysis was performed among the seven studies conducted in the general population. All studies were cross-sectional. As shown in Figure 2, the pooled OR of T2DM in the highest category of urinary NAP metabolite in comparison to the lowest category was 1.52 (95%CI: 1.19, 1.94). Similarly, the pooled OR of T2DM related to urinary FLU metabolite (Figure 3) and urinary PHEN (Figure 4) metabolite were 1.53 (95%CI: 1.36, 1.71) and 1.43 (95%CI: 1.28, 1.60), respectively. Moreover, the pooled OR was more pronounced in urinary ΣOH-PAHs than in 1-OHP (Figure 5, ΣOH-PAHs: 1.49 (95%CI: 1.07, 2.08) vs. 1-OHP: 1.16 (95%CI: 0.91, 1.48).

Quantitative analysis in the occupational population was not performed because only two studies were included. The results for the same types of PAH metabolites in both studies are shown in Figures S5–S8. The results for most PAH metabolites were not significant in the coke oven workers, except for 4-OHPh which was reported to have a dose-dependent association with the risk of T2DM [18].

## 4. Discussion

The current meta-analysis suggests a strong association between the metabolite levels in urinary PAHs and the risk of T2DM in the general population. All of the original studies included in this analysis took OH-PAHs as an internal exposure. In previous experimental studies, researchers directly measured PAHs in the blood and tissues of animals. However, these methods have not been widely used in humans due to their high cost [21]. The most commonly used biomarkers of PAH exposure are urinary PAH metabolites and PAH-DNA adducts. Among them, urinary PAH metabolites have been reported to be strongly correlated with the concentration of environmental PAHs [21]. In addition, there is an increasing trend of using bioaccumulation and exposure-induced biochemical effects to evaluate the impacts of pollutants [6]. Therefore, the measurement of urinary PAH metabolites is a convenient way to reflect environmental PAH exposure.

Different individual PAHs were used to indicate possible sources of pollution. High- molecule-weight PAHs (HMW PAHs), such as pyrene and benzo(a)pyrene, come mainly from pyrogenic sources. LMW PAHs, such as NAP, FLU, and PHEN, come mainly from petrogenic sources [32]. Urinary 1-OHP, the main metabolite of pyrene, is increasingly used as a marker of PAH exposure. In the current analysis, the pooled OR of T2DM associated with 1-OHP was not significant. Although the metabolites of other HMW PAHs were not analyzed in this study, an association between 9-hydroxybenzpyrene and high glucose was reported by Zhang et al. This result was also not statistically significant [29]. However, the current analysis showed that the metabolites of LMW PAHs (2 to 3 rings), such as hydroxynaphthalene, hydroxyfluorene, and hydroxyphenanthrene, significantly increased the risk of T2DM. These results suggest that LMW PAHs may have a greater impact on diabetes compared to HMW PAHs.

Occupational exposure is an important source of exposure to high levels of PAHs. Therefore, two studies conducted in coke oven workers were reviewed. Only Yang et al. showed a dose–response relationship between 4-OHPh and an increased risk of T2DM [18]. There was insufficient evidence to confirm an association between other types of PAH metabolites and diabetes in occupational workers. Cooking is also an important source of PAHs. J Hou et al. found that Chinese women who cooked frequently had a higher risk of T2DM and a higher body burden of urinary OH-PAH levels [33]

Evidence from mechanistic studies supports a number of potential pathways linking PAHs to T2DM. The first mechanism is the chronic activation of inflammation. Several studies have suggested that PAHs play a key role in systemic inflammation. Khalil et al. demonstrated that the environmental contaminant benzo(a)pyrene (BAP) enhanced the effects of a high-fat diet on intestinal inflammation in mice [7]. Alshaarawy et al. later found that exposure to 1-OHP elevated inflammatory markers, including total leukocyte count and high-sensitivity serum CRP [34]. The second mechanism is increased insulin resistance. A panel study in Korean older adults showed a dose–response relationship between insulin resistance and urinary levels of 1-OHP, which may further contribute to disturbed glucose metabolism [9]. In the same year, Hu H et al. also found that exposure to OH-PAHs led to insulin resistance, β-cell dysfunction, and increased prevalence of metabolic syndrome among nondiabetic adults in the United States [35]. The third potential mechanism may be the dysregulation of cytokine release and the disruption of signal transduction pathways [36,37]. Khalil et al. found that long-term exposure to environmental PAHs can increase the risk of T2DM by inducing the production of pro-inflammatory cytokines [7]. Kim et al. also found that PAHs can induce the methylation of insulin receptor substrate 2 and contribute to insulin resistance [38]. Oxidative stress may be the fourth potential mechanism by which PAH exposure leads to the development of T2DM. PAHs have been shown to induce intracellular oxidative stress in pulmonary epithelial cells [39]. In addition, a longitudinal study conducted in Boston showed that urinary PAH metabolite levels were associated with the plasma inflammatory marker CRP and the urinary oxidative stress markers 8-hydroxydeoxyguanosine and 8-isoprostane [40]. This may lead to a deterioration in pancreatic β-cell function in T2DM patients, resulting in reduced insulin synthesis and secretion [41]. Furthermore, PAHs are potent endocrine disruptors with estrogenic activity [4,14], which is one of the pathologies that may potentially increase the risk of T2DM [42].

Humans ingest PAHs in a variety of ways, such as via inhalation, dermal contact, and ingestion [43]. There are various factors that influence how PAHs affect human health. Some important factors include the duration and route of exposure, the volume or concentration of the PAHs to which one is exposed, and the relative toxicity of the PAHs [43]. The effects of PAHs on human health have been widely studied in recent years. In addition to an increased risk of T2DM, PAHs have also been reported to be associated with other diseases, including cardiovascular diseases [44], lung cancer [45,46], prostate cancer [47], osteoporosis [48], and so on. A review of these literatures may provide new ideas for further exploration of the association between PAHs and T2DM in the future. For example, studies of the association between PAHs and cardiovascular disease have been conducted in several countries, including the United States [49,50,51], China [52,53], Korea [54], Sweden [55], Saudi Arabia [56], and Belgium [57]. However, studies of the effects of PAHs on T2DM have only been conducted in the United States, China, and Korea. Therefore, there is a need to expand the geographical scope of the study to reveal whether there are geographical differences in the effects of PAHs on T2DM. In addition, studies exploring the association between PAHs and cardiovascular disease have used a variety of study designs, including time-series [49,50,51], retrospective cohort [58], cross-sectional [59], panel [57], and case-control studies [60]. However, the current epidemiological studies investigating the effect of PAHs on T2DM are all cross-sectional studies. Therefore, more study designs should be adopted in future studies.

Although a broad search strategy was implemented in this study and filters were not applied in the analysis, there were only a few studies on the relationship between urinary PAH metabolites and T2DM and no longitudinal studies that met the inclusion criteria. In the analysis of the current study, a quantitative approach was used to systematically assess the results of the primary studies. The results showed that urinary PAH metabolites levels increased the risk of T2DM. The study provides evidence-based medical support for smoking bans and further reduction in PAH emissions. The current meta-analysis included studies conducted in both developed and developing countries. In terms of generalizability of the evidence, the current study included populations living in developing countries with relatively high levels of air pollution and populations living in developed countries with relatively low levels of air pollution. This may help us to understand the harmful effects of different air pollution components. Although influenced by different developmental status, ethnicity, and pollutant levels, this meta-analysis provides evidence of a positive association between urinary PAH metabolites and T2DM in the general population. Fewer studies have been conducted in occupational workers, but the available evidence suggested a nonsignificant association between multiple PAH metabolites and the risk of diabetes or hyperglycemia. More studies should be conducted in occupational populations, as this may reflect the effects of very high levels of pollutants on the human body. More evidence is needed to improve the environment to which occupational workers are exposed.

Based on the available evidence, future studies should explore the causal relationship between PAH exposure and T2DM and consider the various time-lagged or cumulative effects of PAH exposure. Future studies should quantify the level of PAH exposure for direct comparison with existing evidence. Socioeconomic variables should be considered, as low socioeconomic status is associated with PAH exposure and development of T2DM [61]. Also, individual risk factors such as obesity, sleep, physical activity, nutrition, cigarette smoking, and alcohol drinking should be measured and considered when assessing the health effects of urinary PAH metabolites. Lack of information on these confounding factors may lead to a high risk of bias. When measuring urinary PAH metabolite levels, it is important to adjust for urine dilution. Direct adjustment of creatinine levels may be inappropriate when study outcomes are related to BMI. Furthermore, the adverse health effects of PAHs in humans depend partly on the route of exposure [3], which is determined by their molecular weight. Therefore, future studies could evaluate the impact of LMW PAHs and HMW PAHs on health outcomes separately.

## 5. Conclusions

In recent years, several studies exploring the association between urinary PAH metabolites and the risk of T2DM have been published. The available evidence from primary studies suggests that in the general population, urinary levels of multiple PAH metabolites, including NAP, FLU, PHEN, 1-OHP, and total OH-PAH metabolites are positively associated with the risk of T2DM. In studies of occupational populations, only 4-OHPh is reported to be significantly associated with the risk of T2DM. There is limited existing research on occupational populations, so there were not enough studies for a quantitative synthesis analysis. Therefore, future research needs to focus on occupational populations. Despite the limited studies with a potential risk of bias, PAHs may be a new risk factor for T2DM. High-quality longitudinal studies are expected to advance the understanding of this association.

## Figures and Tables

**Table 1:** Description of the included studies.

Study ID	Location	Study Period (Years)	Population (*n*)	Age (Years)	Urinary PAH Metabolites	Measurement
**General Population**						
Alshaarawy et al., 2014 [21]	USA	2001–2002 2003–2004 2005–2006	3326	20–65	1-OHNa, 2-OHNa, 2-OHFlu, 3-OHFlu, 1-OHPh, 2-OHPh, 3-OHPh, and 1-OHP	Capillary gas chromatography combined with high-resolution mass spectrometry (GC-HRMS)
Yang et al., 2014 [24]	China	2011	3092	18–90	1-OHP, 1-OHNa, 2-OHNa, 2-OHFlu, 9-OHFlu, 1-OHPh, 2-OHPh, 3-OHPh, 4-OHPh, 9-OHPh, 6-OHChr, and 3-OHBaP (6-OHChr and 3-OHBaP were below the limits of quantification)	Gas chromatographye mass spectrometry (GC/MS)
Ranjbar et al., 2015 [22]	USA	2001–2008	4765	≥20	1-OHNa, 2-OHNa, 2-OHFlu, 3-OHFlu, 1-OHPh, 2-OHPh, 3-OHPh, and 1-OHP	Capillary gas chromatography combined with high-resolution mass spectrometry (GC-HRMS)
Smith et al., 2018 [20]	USA	2005–2014	8664	adults and children	1-OHNa, 2-OHNa, 2-OHFlu, 3-OHFlu, 9-OHFlu, 1-OHPh, 2-OHPh, 3-OHPh, and 1-OHP	Gas and liquid chromatography-tandem mass spectrometry
Cheng et al., 2021 [25]	China	2011.04~05, 2012.05	3031	18–80	1-OHNa, 2-OHNa, 2-OHFlu, 9-OHFlu, 1-OHPh, 2-OHPh, 3-OHPh, 4-OHPh, 9-OHPh, and 1-OHP.	Agilent 5975B/6890 N GCeMS System (Agilent, Santa Clara, CA, USA)
Nam et al., 2020 [28]	Korea	2012–2014	6478	≥19	1-OHP, 2-OHNa, 1-OHPh, and 2-OHFlu	Gas chromatography-mass spectrometry (Clarus 680T, PerkinElmer, Waltham, MA, USA)
Lee et al., 2022 [23]	Korea	2015–2017	OH-PAHs except for 1-OHPhe (*n* = 3751), all OH-PAHs (*n* = 3754)	≥19	1-OHP, 2-OHNa, 1-OHPh, and 2-OHFlu	Gas chromatography–mass spectrometry
**Occupational workers (coke oven workers)**						
Yang et al., 2017 [18]	China	2010–2014	1472	47.6 ± 7.1 for diabetic patients	1-OHNa, 2-OHNa, 2-OHFlu, 9-OHFlu, 1-OHPh, 2-OHPh, 3-OHPh, 4-OHPh, 9-OHPh, 1-OHP, 6-OHChr, 3-OHBaP	Gas chromatography-mass spectrometry (GC-MS, Agilent, Santa Clara, CA, USA)
Zhang et al., 2020 [29]	China	2017	682	31–48	1-OHNa, 2-OHNa, 2-OHFlu, 3-OHFlu, 1-OHPh, 2-OHPh, 9-OHPh, 1-OHP, 3-OHChr, 6-OHChr and, 9-OHBaP	High performance liquid chromatography mass spectrometry (HPLC-MS)
**Study ID**		**Definition of T2DM**		**Adjusting Method for Urine Dilution**		**Adjusted Confounder**
**General Population**						
Alshaarawy et al., 2014 [21]		HbA1c level ≥ 6.5% (39 mmol/mol), a self-reported physician diagnosis of diabetes, or current use of oral hypoglycemic medication or insulin.		Urinary levels of OH-PAH (ng/L) were divided by urinary creatinine level (mg/dL) multiplied by 0.01, that is, (ng/L) ÷ (mg/dL × 0.01), and expressed as nanogram per gram of creatinine (ng/g creatinine)		age, sex, ethnicity, poverty–income ratio, alcohol drinking, BMI, total cholesterol and serum cotinine
Yang et al., 2014 [24]		FBG ≥ 7.0 mmol/L, self-reported physician-diagnosed diabetes, or taking oral hypoglycemic medication or insulin.		Valid urinary PAHs metabolites concentrations were calibrated by levels of urinary creatinine and calculated as nmol/mmol creatinine.		age, sex, BMI, smoking status, alcohol consumption, physical activity, education, family history of diabetes, total cholesterol, and triglycerides.
Ranjbar et al., 2015 [22]		FPG ≥ 7 mmol/L, HbA1c ≥ 6.5%, doctor diagnosed T2DM, taking diabetes medication, or were taking insulin.		Urinary creatinine was adjusted in logistic regressions model.		age, sex, poverty index ratio, ethnicity, BMI, smoking status and urinary creatinine
Smith et al., 2018 [20]		HbA1c ≥ 6.5%, self-reported diagnosis of diabetes by a physician, and/or self-reported insulin use.		Exposure variables were corrected for urinary creatinine in all analyses by dividing each of the PAHs (ng/L) by urinary creatinine (mg/dL) and multiplying by 0.01 to result in nanograms of PAHs per gram of creatinine (ng/g)		age, sex, race, poverty-income ratio, and serum cotinine.
Cheng et al., 2021 [25]		FPG ≥ 7.0 mmol/L, self-reported diagnosis of diabetes, using oral antidiabetic agents or insulin		The levels of urinary creatinine (Cr) were used to calibrate each valid urinary PAH metabolite concentrations.		age, sex, BMI, drug usage, smoking status, drinking status, physical activity, family income, city, and family history of diabetes
Nam et al., 2020 [28]		Self-report of physician-diagnosed diabetes mellitus or the use of oral hypoglycemics or insulin.		All urinary PAH levels were adjusted for the urinary creatinine levels.		sex, age, BMI, household income, alcohol consumption, physical activity, log-transformed urinary creatinine and cotinine, and menopausal status (in women)
Lee et al., 2022 [23]		Those who reported using DM medication were assumed to have T2DM.		Covariate-adjusted standardized chemical measure = Chemical concentration × the predicted Cr level/the measured Cr level; A conventional Cr adjustment was also applied		age, sex, BMI, cigarette smoking, alcohol drinking, education, and exercise
**Occupational workers (coke oven workers)**						
Yang et al., 2017 [18]		Receiving diabetes medications, or FBG ≥ 7.0 mmol/L, or self-reported physician-diagnosed diabetes.		Valid urinary PAH metabolite concentrations were calibrated by levels of urinary creatinine and expressed as micrograms per millimole creatinine (mg/mmol creatinine).		working years, sex, BMI, smoking status, drinking status, physical activity, education, workshift, work sites, family history of diabetes, total cholesterol, and triglycerides
Zhang et al., 2020 [29]		High FBG levels as a component of MetS: FBG > 6.10 mmol/L or current use of medication to treat hyperglycaemia.		Valid urine concentrations of PAH metabolites were adjusted using urine gravity.		sex, age, smoking, drinking, cooking fumes, eating habits, BMI, and other PAH metabolites

## Data Availability

The data presented in this study are available on request from the corresponding author.

## Supplementary Materials

The following supporting information can be downloaded at: https://www.mdpi.com/article/10.3390/ijerph19137605/s1, Figure S1: Funnel plot for the association between urinary NAP metabolites and diabetes. *p*-value of Begg’s Test: 0.0746, *p*-value of Egger’s test: 0.1757; Figure S2: Funnel plot for the association between urinary FLU metabolites and diabetes. *p*-value of Begg’s Test: 0.7143, *p*-value of Egger’s test: 0.7238; Figure S3: Funnel plot for the association between urinary PHEN metabolites and diabetes. *p*-value of Begg’s Test: 0.6074, *p*-value of Egger’s test: 0.4144; Figure S4: Funnel plot for the association between urinary 1-OHP, ΣOH-PAH metabolites and diabetes. *p*-value of Begg’s Test: 0.4106, *p*-value of Egger’s test: 0.3077; Figure S5: The association between urinary NAP metabolites and risk of T2DM in coke oven workers; Figure S6: The association between urinary FLU metabolites and risk of T2DM in coke oven workers; Figure S7: The association between urinary PHEN metabolites and risk of T2DM in coke oven workers; Figure S8: The association between urinary 1-OHP, ΣOH-PAHs metabolites and risk of T2DM in coke oven workers.

## Abbreviations

PAHs	polycyclic aromatic hydrocarbons
OH-PAHs	mono-hydroxylated PAHs
T2DM	type 2 diabetes
LMW	low molecule weight
HMW	high molecule weight
PM	particulate matter
1-OHNa	1-hydroxynaphthalene
2-OHNa	2-hydroxynaphthalene
2-OHFlu	2-hydroxyfluorene
3-OHFlu	3-hydroxyfluorene
9-OHFlu	9-hydroxyfluorene
1-OHPh	1-hydroxyphenanthrene
2-OHPh	2-hydroxyphenanthrene
3-OHPh	3-hydroxyphenanthrene
4-OHPh	4-hydroxyphenanthrene
9-OHPh	9-hydroxyphenanthrene
1-OHP	1-hydroxypyrene
BAP	benzo(a)pyrene
CIs	confidence intervals
OR	odds ratio
RR	relative risk
HR	hazard ratio
8-OHdG	8-hydroxydeoxyguanosine
SES	socioeconomic status

## References

[1] Liu C., Chen R., Sera F., Vicedo-Cabrera A.M., Guo Y., Tong S., Coelho M., Saldiva P.H.N., Lavigne E., Matus P. (2019). Ambient Particulate Air Pollution and Daily Mortality in 652 Cities. N. Engl. J. Med..

[2] Cohen A.J., Brauer M., Burnett R., Anderson H.R., Frostad J., Estep K., Balakrishnan K., Brunekreef B., Dandona L., Dandona R. (2017). Estimates and 25-year trends of the global burden of disease attributable to ambient air pollution: An analysis of data from the Global Burden of Diseases Study 2015. Lancet.

[3] Kim K.-H., Jahan S.A., Kabir E., Brown R.J.C. (2013). A review of airborne polycyclic aromatic hydrocarbons (PAHs) and their human health effects. Environ. Int..

[4] Zhang Y., Dong S., Wang H., Tao S., Kiyama R. (2016). Biological impact of environmental polycyclic aromatic hydrocarbons (ePAHs) as endocrine disruptors. Environ. Pollut..

[5] Gao P., da Silva E., Hou L., Denslow N.D., Xiang P., Ma L.Q. (2018). Human exposure to polycyclic aromatic hydrocarbons: Metabolomics perspective. Environ. Int..

[6] Srogi K. (2007). Monitoring of environmental exposure to polycyclic aromatic hydrocarbons: A review. Environ. Chem. Lett..

[7] Khalil A., Villard P.H., Dao M.A., Burcelin R., Champion S., Fouchier F., Savouret J.F., Barra Y., Seree E. (2010). Polycyclic aromatic hydrocarbons potentiate high-fat diet effects on intestinal inflammation. Toxicol. Lett..

[8] Everett C.J., King D.E., Player M.S., Matheson E.M., Post R.E., Mainous A.G. (2010). Association of urinary polycyclic aromatic hydrocarbons and serum C-reactive protein. Environ. Res..

[9] Choi Y.H., Kim J.H., Hong Y.C. (2015). Sex-dependent and body weight-dependent associations between environmental PAHs exposure and insulin resistance: Korean urban elderly panel. J. Epidemiol. Community Health.

[10] Gato W.E., Hunter D.A., Whitby S.L., Mays C.A., Yau W. (2016). Investigating susceptibility to diabetes using features of the adipose tissue in response to in utero polycyclic aromatic hydrocarbons exposure. Diabetes Metab. J..

[11] Guo H., Huang K., Zhang X., Zhang W., Guan L., Kuang D., Deng Q., Deng H., Zhang X., He M. (2014). Women are more susceptible than men to oxidative stress and chromosome damage caused by polycyclic aromatic hydrocarbons exposure. Environ. Mol. Mutagen..

[12] Archibong A.E., Inyang F., Ramesh A., Greenwood M., Nayyar T., Kopsombut P., Hood D.B., Nyanda A.M. (2002). Alteration of pregnancy related hormones and fetal survival in F-344 rats exposed by inhalation to benzo(a)pyrene. Reprod. Toxicol..

[13] Makaji E., Raha S., Wade M.G., Holloway A.C. (2011). Effect of environmental contaminants on Beta cell function. Int. J. Toxicol..

[14] Idowu O., Semple K.T., Ramadass K., O’Connor W., Hansbro P., Thavamani P. (2019). Beyond the obvious: Environmental health implications of polar polycyclic aromatic hydrocarbons. Environ. Int..

[15] Yang X., Tao S., Peng J., Zhao J., Li S., Wu N., Wen Y., Xue Q., Yang C.X., Pan X.F. (2021). High-sensitivity C-reactive protein and risk of type 2 diabetes: A nationwide cohort study and updated meta-analysis. Diabetes Metab. Res. Rev..

[16] Lamichhane D.K., Leem J.H., Kim H.C., Lee J.Y., Park M.S., Jung D.Y., Ko J.K., Ha M., Kim Y., Hong Y.C. (2016). Impact of prenatal exposure to polycyclic aromatic hydrocarbons from maternal diet on birth outcomes: A birth cohort study in Korea. Public Health Nutr..

[17] Li Y., Qi Q., Workalemahu T., Hu F.B., Qi L. (2012). Birth weight, genetic susceptibility, and adulthood risk of type 2 diabetes. Diabetes Care.

[18] Yang L., Yan K., Zeng D., Lai X., Chen X., Fang Q., Guo H., Wu T., Zhang X. (2017). Association of polycyclic aromatic hydrocarbons metabolites and risk of diabetes in coke oven workers. Environ. Pollut..

[19] Khosravipour M., Khosravipour H. (2020). The association between urinary metabolites of polycyclic aromatic hydrocarbons and diabetes: A systematic review and meta-analysis study. Chemosphere.

[20] Stallings-Smith S., Mease A., Johnson T.M., Arikawa A.Y. (2018). Exploring the association between polycyclic aromatic hydrocarbons and diabetes among adults in the United States. Environ. Res..

[21] Alshaarawy O., Zhu M., Ducatman A.M., Conway B., Andrew M.E. (2014). Urinary polycyclic aromatic hydrocarbon biomarkers and diabetes mellitus. Occup. Environ. Med..

[22] Ranjbar M., Rotondi M.A., Ardern C.I., Kuk J.L. (2015). Urinary Biomarkers of Polycyclic Aromatic Hydrocarbons Are Associated with Cardiometabolic Health Risk. PLoS ONE.

[23] Lee I., Park H., Kim M.J., Kim S., Choi S., Park J., Cho Y.H., Hong S., Yoo J., Cheon G.J. (2022). Exposure to polycyclic aromatic hydrocarbons and volatile organic compounds is associated with a risk of obesity and diabetes mellitus among Korean adults: Korean National Environmental Health Survey (KoNEHS) 2015–2017. Int. J. Hyg. Environ. Health.

[24] Yang L., Zhou Y., Sun H., Lai H., Liu C., Yan K., Yuan J., Wu T., Chen W., Zhang X. (2014). Dose-response relationship between polycyclic aromatic hydrocarbon metabolites and risk of diabetes in the general Chinese population. Environ. Pollut..

[25] Cheng M., Zhou Y., Wang B., Mu G., Ma J., Zhou M., Wang D., Yang M., Cao L., Xie L. (2021). IL-22: A potential mediator of associations between urinary polycyclic aromatic hydrocarbon metabolites with fasting plasma glucose and type 2 diabetes. J. Hazard. Mater..

[26] Viau M.B. (1999). Urinary 1-hydroxypyrene as a biomarker of exposure to polycyclic aromatic hydrocarbons: Biological monitoring strategies and methodology for determining biological exposure indices for various work environments. Biomarkers.

[27] Liu B., Feng W., Wang J., Li Y., Han X., Hu H., Guo H., Zhang X., He M. (2016). Association of urinary metals levels with type 2 diabetes risk in coke oven workers. Environ. Pollut..

[28] Nam Y.J., Kim S.H. (2020). Association of Urinary Polycyclic Aromatic Hydrocarbons and Diabetes in Korean Adults: Data from the Korean National Environmental Health Survey Cycle 2 (2012–2014). Diabetes Metab. Syndr. Obes. Targets Ther..

[29] Zhang B., Pan B., Zhao X., Fu Y., Li X., Yang A., Li Q., Dong J., Nie J., Yang J. (2020). The interaction effects of smoking and polycyclic aromatic hydrocarbons exposure on the prevalence of metabolic syndrome in coke oven workers. Chemosphere.

[30] Barr D.B., Wilder L.C., Caudill S.P., Gonzalez A.J., Needham L.L., Pirkle J.L. (2005). Urinary creatinine concentrations in the U.S. population: Implications for urinary biologic monitoring measurements. Environ. Health Perspect..

[31] Wang Y., Wong L.Y., Meng L., Pittman E.N., Trinidad D.A., Hubbard K.L., Etheredge A., Del Valle-Pinero A.Y., Zamoiski R., van Bemmel D.M. (2019). Urinary concentrations of monohydroxylated polycyclic aromatic hydrocarbons in adults from the U.S. Population Assessment of Tobacco and Health (PATH) Study Wave 1 (2013–2014). Environ. Int..

[32] Patel A.B., Shaikh S., Jain K.R., Desai C., Madamwar D. (2020). Polycyclic Aromatic Hydrocarbons: Sources, Toxicity, and Remediation Approaches. Front. Microbiol..

[33] Hou J., Sun H., Zhou Y., Zhang Y., Yin W., Xu T., Cheng J., Chen W., Yuan J. (2018). Environmental exposure to polycyclic aromatic hydrocarbons, kitchen ventilation, fractional exhaled nitric oxide, and risk of diabetes among Chinese females. Indoor Air.

[34] Alshaarawy O., Zhu M., Ducatman A., Conway B., Andrew M.E. (2013). Polycyclic aromatic hydrocarbon biomarkers and serum markers of inflammation. A positive association that is more evident in men. Environ. Res..

[35] Hu H., Kan H.D., Kearney G.D., Xu X.H. (2015). Associations between exposure to polycyclic aromatic hydrocarbons and glucose homeostasis as well as metabolic syndrome in nondiabetic adults. Sci. Total Environ..

[36] Niu X., Ho S.S.H., Ho K.F., Huang Y., Sun J., Wang Q., Zhou Y., Zhao Z., Cao J. (2017). Atmospheric levels and cytotoxicity of polycyclic aromatic hydrocarbons and oxygenated-PAHs in PM2.5 in the Beijing-Tianjin-Hebei region. Environ. Pollut..

[37] Yang B., Deng Q., Zhang W., Feng Y., Dai X., Feng W., He X., Huang S., Zhang X., Li X. (2016). Exposure to Polycyclic Aromatic Hydrocarbons, Plasma Cytokines, and Heart Rate Variability. Sci. Rep..

[38] Kim Y.H., Lee Y.S., Lee D.H., Kim D.S. (2016). Polycyclic aromatic hydrocarbons are associated with insulin receptor substrate 2 methylation in adipose tissues of Korean women. Environ. Res..

[39] Zhou Q., Liu B., Chen Y., Han X., Wei X., Zhu Y., Zhou X., Chen J. (2017). Characterization of PAHs in size-fractionated submicron atmospheric particles and their association with the intracellular oxidative stress. Chemosphere.

[40] Ferguson K.K., McElrath T.F., Pace G.G., Weller D., Zeng L., Pennathur S., Cantonwine D.E., Meeker J.D. (2017). Urinary Polycyclic Aromatic Hydrocarbon Metabolite Associations with Biomarkers of Inflammation, Angiogenesis, and Oxidative Stress in Pregnant Women. Environ. Sci. Technol..

[41] Robertson R.P. (2006). Oxidative stress and impaired insulin secretion in type 2 diabetes. Curr. Opin. Pharmacol..

[42] Alonso-Magdalena P., Morimoto S., Ripoll C., Fuentes E., Nadal A. (2006). The estrogenic effect of bisphenol A disrupts pancreatic beta-cell function in vivo and induces insulin resistance. Environ. Health Perspect..

[43] Mallah M.A., Changxing L., Mallah M.A., Noreen S., Liu Y., Saeed M., Xi H., Ahmed B., Feng F., Mirjat A.A. (2022). Polycyclic aromatic hydrocarbon and its effects on human health: An overeview. Chemosphere.

[44] Mallah M.A., Mallah M.A., Liu Y., Xi H., Wang W., Feng F., Zhang Q. (2021). Relationship Between Polycyclic Aromatic Hydrocarbons and Cardiovascular Diseases: A Systematic Review. Front. Public Health.

[45] Stading R., Gastelum G., Chu C., Jiang W., Moorthy B. (2021). Molecular mechanisms of pulmonary carcinogenesis by polycyclic aromatic hydrocarbons (PAHs): Implications for human lung cancer. Semin. Cancer Biol..

[46] Moorthy B., Chu C., Carlin D.J. (2015). Polycyclic aromatic hydrocarbons: From metabolism to lung cancer. Toxicol. Sci..

[47] Barul C., Parent M.E. (2021). Occupational exposure to polycyclic aromatic hydrocarbons and risk of prostate cancer. Environ. Health.

[48] Duan W., Meng X., Sun Y., Jia C. (2018). Association between polycyclic aromatic hydrocarbons and osteoporosis: Data from NHANES, 2005–2014. Arch. Osteoporos..

[49] Shiue I. (2015). Are urinary polyaromatic hydrocarbons associated with adult hypertension, heart attack, and cancer? USA NHANES, 2011–2012. Environ. Sci. Pollut. Res. Int..

[50] Xu X., Cook R.L., Ilacqua V.A., Kan H., Talbott E.O., Kearney G. (2010). Studying associations between urinary metabolites of polycyclic aromatic hydrocarbons (PAHs) and cardiovascular diseases in the United States. Sci. Total Environ..

[51] Clark J.D., Serdar B., Lee D.J., Arheart K., Wilkinson J.D., Fleming L.E. (2012). Exposure to polycyclic aromatic hydrocarbons and serum inflammatory markers of cardiovascular disease. Environ. Res..

[52] Cao L., Wang D., Zhu C., Wang B., Cen X., Chen A., Zhou H., Ye Z., Tan Q., Nie X. (2020). Polycyclic aromatic hydrocarbon exposure and atherosclerotic cardiovascular disease risk in urban adults: The mediating role of oxidatively damaged DNA. Environ. Pollut..

[53] Yang L., Guo W., Zeng D., Ma L., Lai X., Fang Q., Guo H., Zhang X. (2019). Heart rate variability mediates the association between polycyclic aromatic hydrocarbons exposure and atherosclerotic cardiovascular disease risk in coke oven workers. Chemosphere.

[54] Lee T.W., Kim D.H., Ryu J.Y. (2020). Association between urinary polycyclic aromatic hydrocarbons and hypertension in the Korean population: Data from the Second Korean National Environmental Health Survey (2012–2014). Sci. Rep..

[55] Alhamdow A., Lindh C., Albin M., Gustavsson P., Tinnerberg H., Broberg K. (2017). Early markers of cardiovascular disease are associated with occupational exposure to polycyclic aromatic hydrocarbons. Sci. Rep..

[56] Trasande L., Urbina E.M., Khoder M., Alghamdi M., Shabaj I., Alam M.S., Harrison R.M., Shamy M. (2015). Polycyclic aromatic hydrocarbons, brachial artery distensibility and blood pressure among children residing near an oil refinery. Environ. Res..

[57] Jacobs L., Buczynska A., Walgraeve C., Delcloo A., Potgieter-Vermaak S., Van Grieken R., Demeestere K., Dewulf J., Van Langenhove H., De Backer H. (2012). Acute changes in pulse pressure in relation to constituents of particulate air pollution in elderly persons. Environ. Res..

[58] Burstyn I., Kromhout H., Partanen T., Svane O., Langard S., Ahrens W., Kauppinen T., Stucker I., Shaham J., Heederik D. (2005). Polycyclic aromatic hydrocarbons and fatal ischemic heart disease. Epidemiology.

[59] Feng Y., Sun H., Song Y., Bao J., Huang X., Ye J., Yuan J., Chen W., Christiani D.C., Wu T. (2014). A community study of the effect of polycyclic aromatic hydrocarbon metabolites on heart rate variability based on the Framingham risk score. Occup. Environ. Med..

[60] Li N., Mu Y., Liu Z., Deng Y., Guo Y., Zhang X., Li X., Yu P., Wang Y., Zhu J. (2018). Assessment of interaction between maternal polycyclic aromatic hydrocarbons exposure and genetic polymorphisms on the risk of congenital heart diseases. Sci. Rep..

[61] Wang L., Hou J., Hu C., Zhou Y., Sun H., Zhang J., Li T., Gao E., Wang G., Chen W. (2019). Mediating factors explaining the associations between polycyclic aromatic hydrocarbons exposure, low socioeconomic status and diabetes: A structural equation modeling approach. Sci. Total Environ..

