# Supplementary material for: The Association between Urinary Polycyclic Aromatic Hydrocarbons Metabolites and Type 2 Diabetes Mellitus
Source: Int J Environ Res Public Health. 2022 Jun 22;19(13):7605. doi: 10.3390/ijerph19137605 (PMC9265723; doi:10.3390/ijerph19137605)
Supplement: Supplementary file 1 [file ijerph-19-07605-s001.zip › ijerph-1723828-supplementary.pdf]

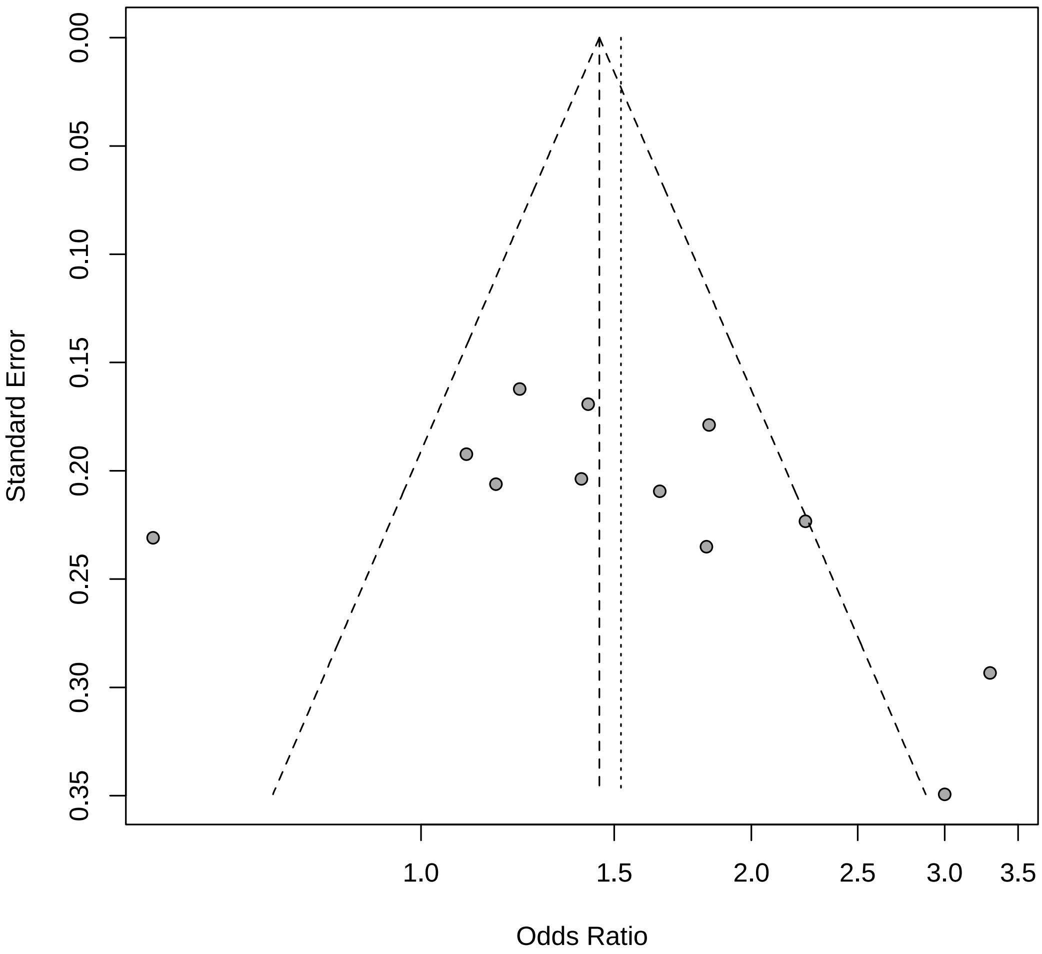

Figure S1. Funnel plot for the association between urinary NAP metabolites and diabetes. P-value of Begg's Test: 0.0746, P-value of Egger's test: 0.1757

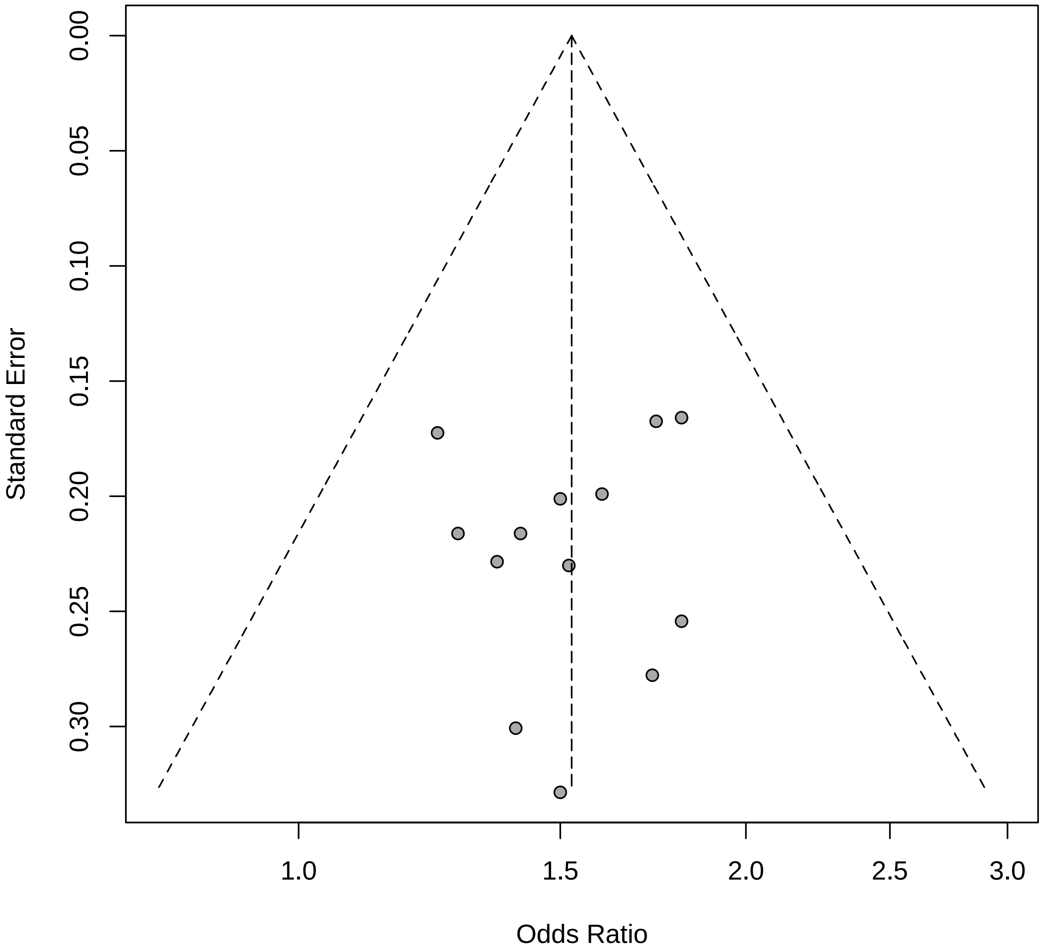

Figure S2. Funnel plot for the association between urinary FLU metabolites and diabetes. P-value of Begg's Test: 0.7143, P-value of Egger's test: 0.7238

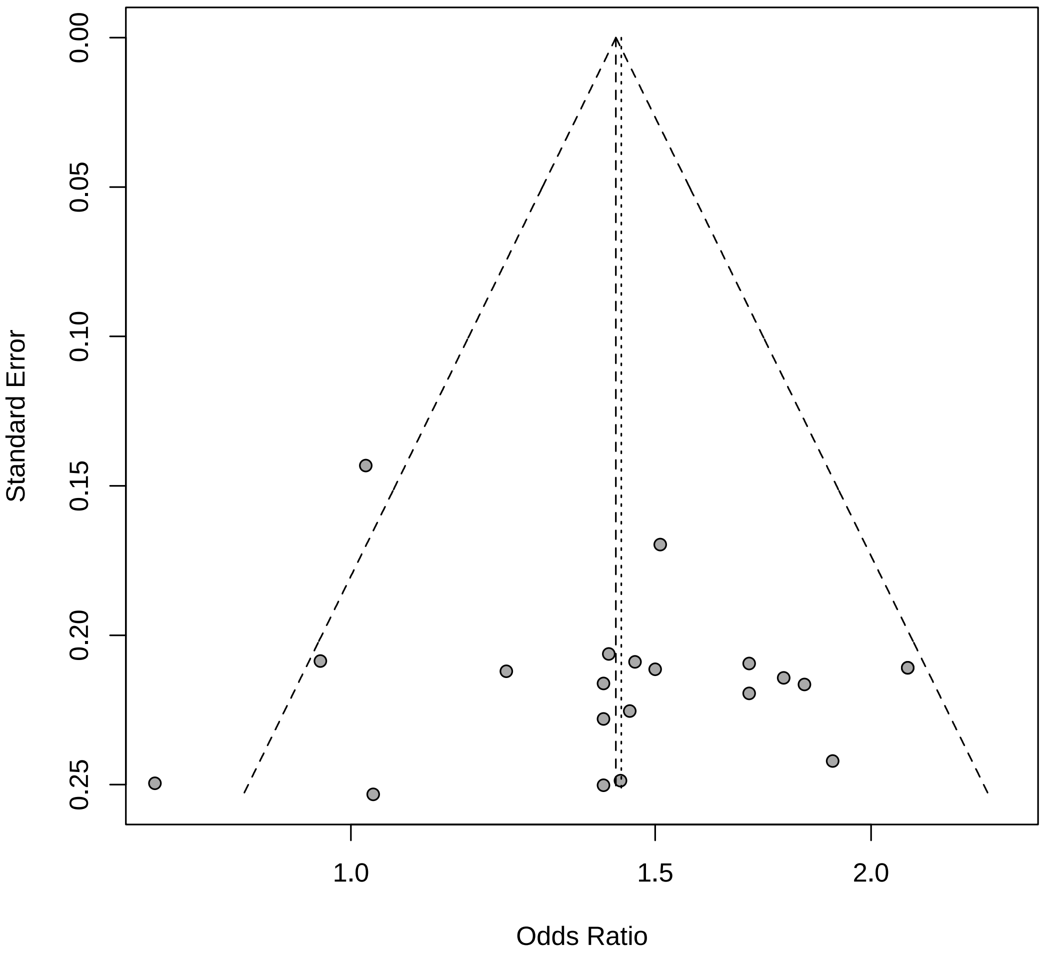

Figure S3. Funnel plot for the association between urinary PHEN metabolites and diabetes. P-value of Begg's Test: 0.6074, P-value of Egger's test: 0.4144

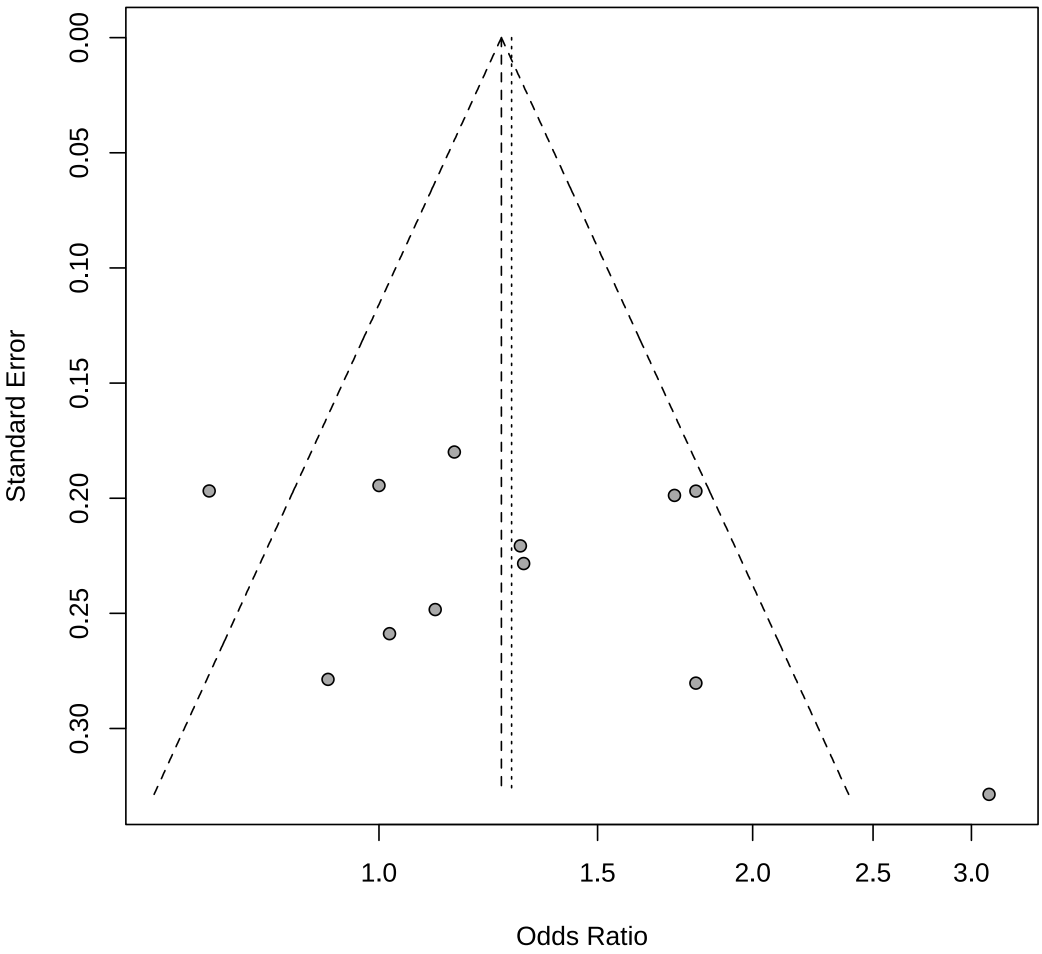

Figure S4. Funnel plot for the association between urinary 1-OHP, ΣOH-PAH metabolites and diabetes. P-value of Begg's Test: 0.4106, P-value of Egger's test: 0.3077

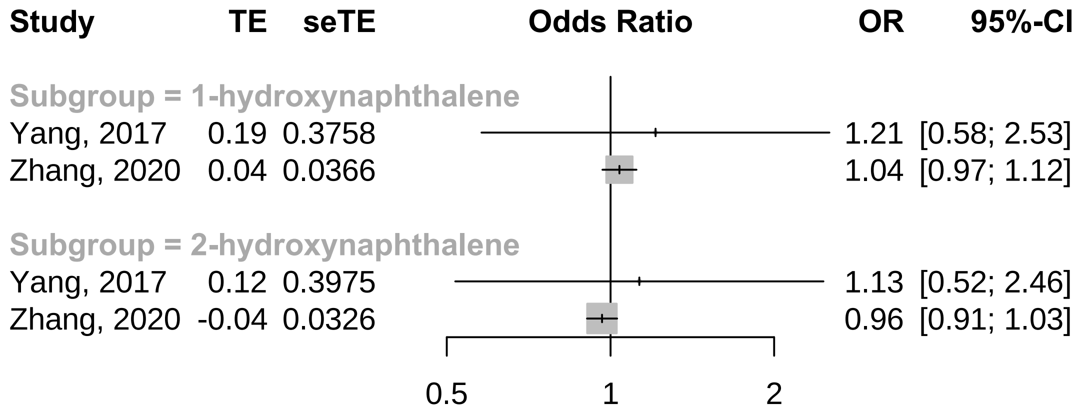

Figure S5. The association between urinary NAP metabolites and risk of T2DM in coke oven workers

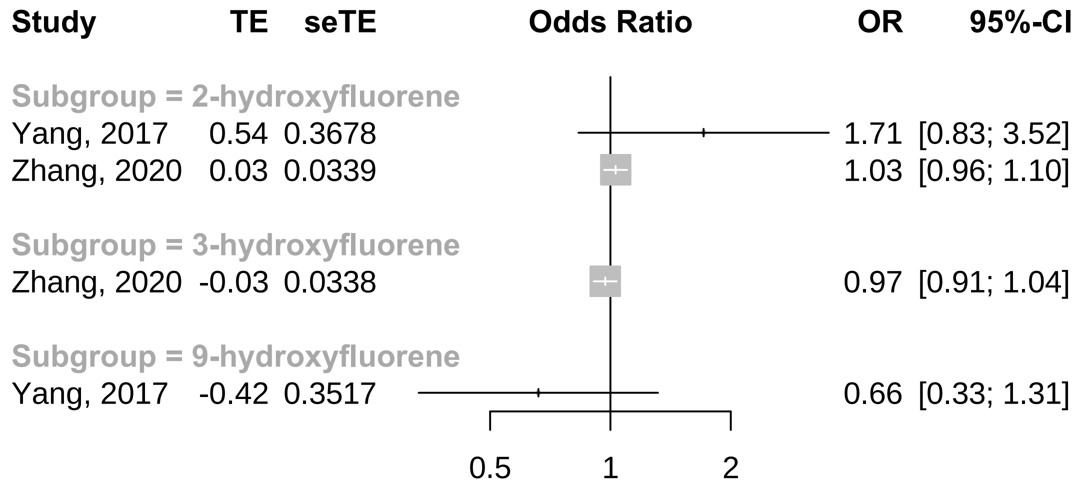

Figure S6. The association between urinary FLU metabolites and risk of T2DM in coke oven workers

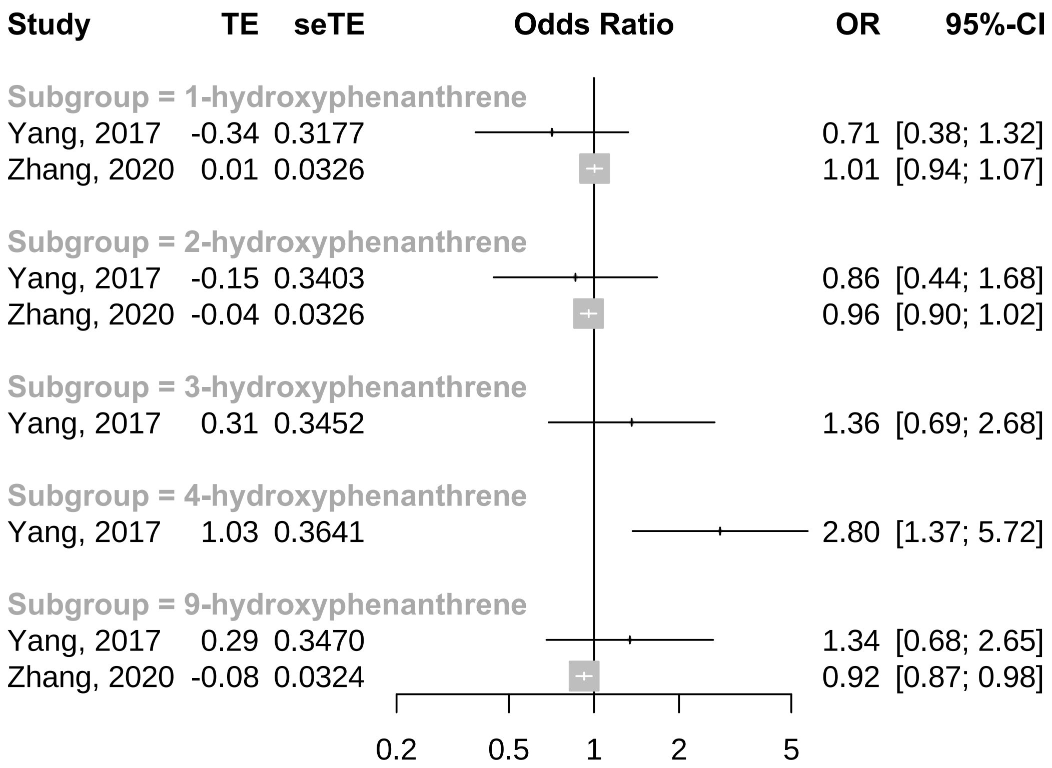

Figure S7. The association between urinary PHEN metabolites and risk of T2DM in coke oven workers

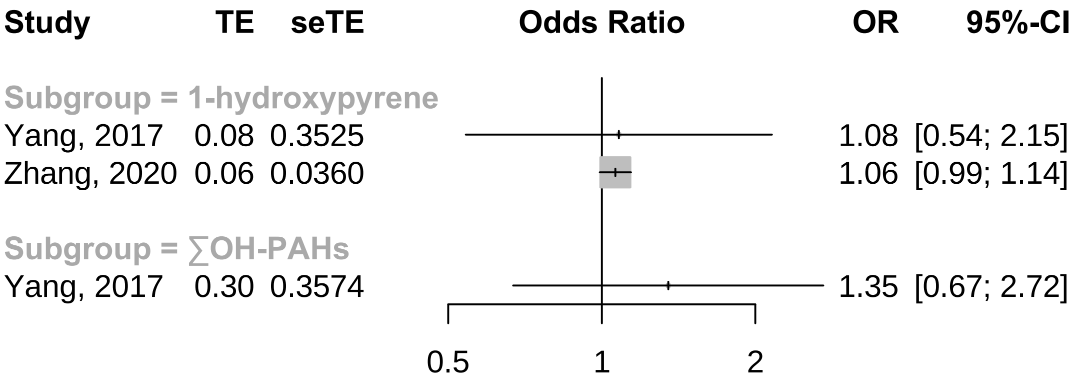

Figure S8. The association between urinary 1-OHP, ΣOH-PAHs metabolites and risk of T2DM in coke oven workers
